# A case of hypereosinophilia with digital ischaemia successfully treated with intravenous prostaglandin E_1_

**DOI:** 10.1002/ski2.32

**Published:** 2021-05-03

**Authors:** M. Kaga, H. Takeuchi, K. Iwahara, H. Ihara

**Affiliations:** ^1^ Division of Dermatology Koto Hospital Tokyo Japan; ^2^ Department of Dermatology and Allergology Faculty of Medicine, Graduate School of Medicine, Juntendo University Tokyo Japan; ^3^ Division of Respiratory Medicine Koto Hospital Tokyo Japan; ^4^ Department of Respiratory Medicine Faculty of Medicine, Graduate School of Medicine, Juntendo University Tokyo Japan

## Abstract

In the therapeutic management of eosinophilic disorder, it is important to prevent hypereosinophilia (HE)‐related organ damage even in the process of diagnosis. We describe here a unique clinical and histopathological findings of the patient with HE accompanied with digital ischaemia. Treatment with intravenous prostaglandin E_1_ was essential for digital ischaemia in our case while benralizumab, humanized monoclonal antibody against interleukin‐5 receptorα, did not affect. Our case suggests an earlier intervention for digital ischaemia in the therapeutic management of eosiniphilic disorder.

## Graphical abstract

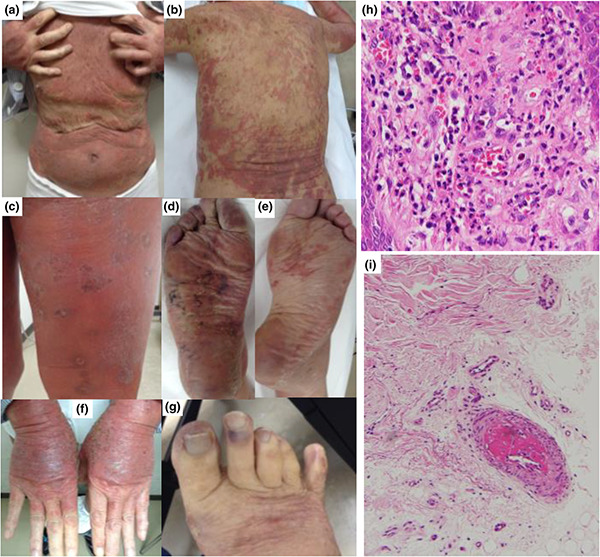

We describe here an unique clinical and histopathological findings of the patient with HE accompanied with digital ischemia. Treatment with intravenous prostaglandin E1 was essential for digital ischemia in our case while benralizumab, humanized monoclonal antibody against interleukin‐5 receptor α, did not affect. The present case highlights the importance of earlier intervention for digital ischemia in the therapeutic management of HE/HES.

## 1 INTRODUCTION

The criterion for hypereosinophilia (HE) is suggested to be a peripheral blood eosinophil count greater than 1.5 × 10^9^/L on two examinations with a minimum interval of 4 weeks.^1^ Idiopathic hypereosinophilic syndrome (HES), HE accompanied with organ damage and/or dysfunction attributable to tissue HE, is diagnosed after exclusion of myeloid/lymphoid neoplasm and secondary causes of eosinophilia, such as infection, allergy/atopy.^1^, ^2^ The diagnostic process to prevent HE‐related organ damage is thus often challenging.

## 2 Case report

**FIGURE 1:**
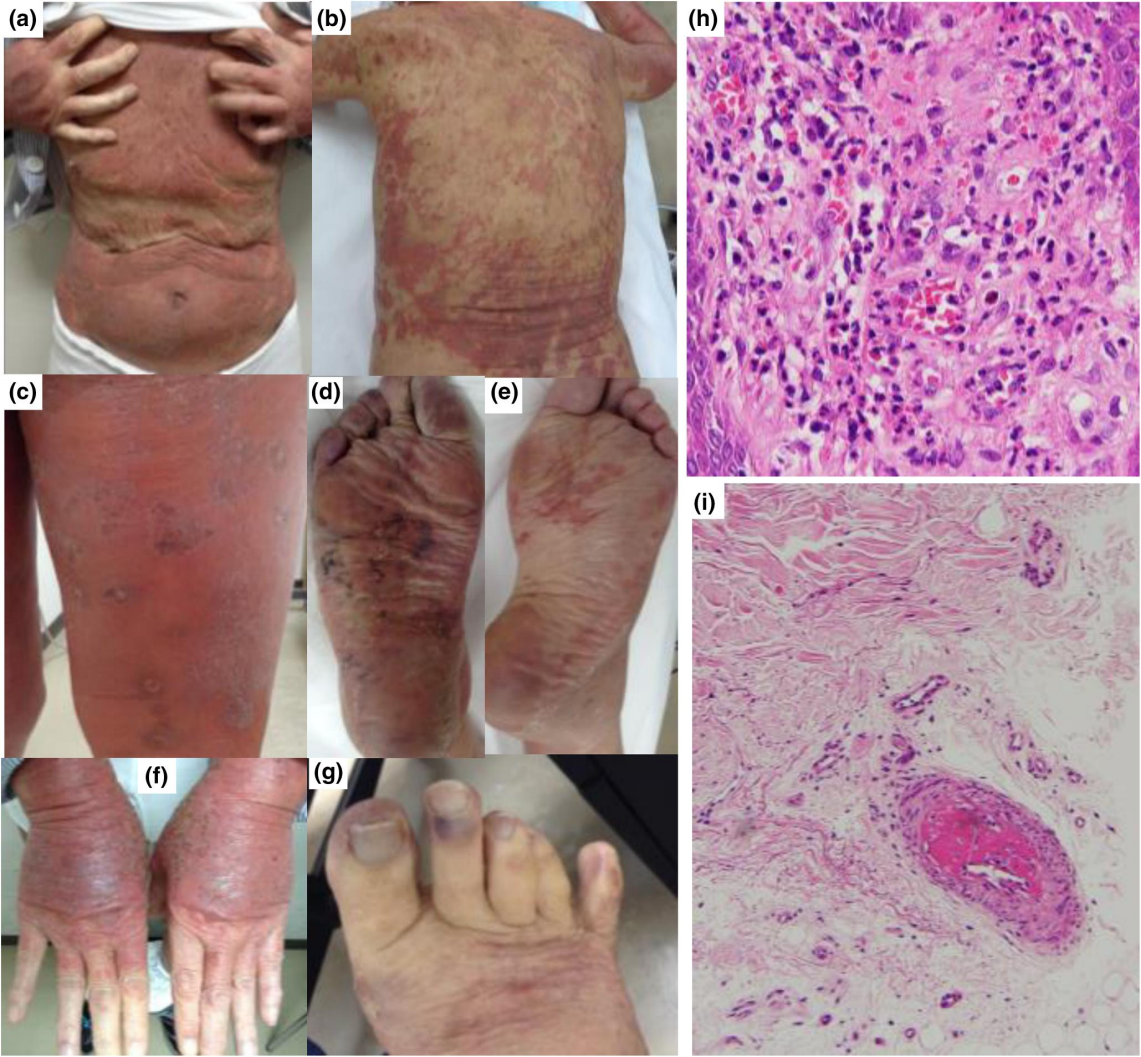
Clinical manifestations on the trunk and right hallux for the patient on admission. Erythroderma and multiple edge‐elevated erythema are observed on the face, head, trunk (a, b) and limb (c, f). Non‐palpable purpura, partly reticulated, are apparent on both soles (d, e) and the right toes with mild pain (g). Skin biopsy from the waist shows diffuse infiltration of the upper dermis by eosinophils and lymphocytes (h). Skin biopsy from the right hallux shows microthrombi (i)

The patient was an 85‐year‐old Japanese man with hypertension, eosinophilic chronic sinusitis, chronic eczema and a 25‐year history of bronchial asthma. From 4 months earlier, he had experienced refractory episodes of pruritic dermatitis that were suspected to represent drug eruption, based on positive lymphocyte transformation test results for clarithromycin, carbocisteine, tipepidine hibenzate, irbesartan, naftopidil and montelukast sodium, each of which he had been taking for several days or weeks. Although these drugs were not continued, his skin condition aggravated to erythroderma together with oedema of lower limbs. Non‐palpable purpura was also observed on the right hallux, right second toe and both soles with mild pain (Figure 1a–g). No fever, organ failure or neuritis was evident.

## 3 Diagnosis and treatment

Eosinophil counts at an interval of 4 weeks in this case were 1.93 × 10^9^/L and 1.82 × 10^9^/L, respectively, fulfilling the diagnostic criteria for HE. Parasite infection was ruled out because he had no bowel symptoms and no current history of travelling abroad. Myeloid/lymphoid neoplasms were ruled out from bone marrow examination. We also could rule out lymphocyte‐variant HES, which is a CD3^−^CD4^+^ variant of HES charactered by an abnormal T‐lymphocyte population inducing HE via eosinophilopoetic cytokines,^3^, ^4^ by flow cytometry on peripheral blood. Skin biopsy taken from the waist showed infiltration of lymphocytes and eosinophils (Figure 1h). His pre‐existing condition such as bronchial asthma and eosinophilic chronic sinusitis strongly indicated the case as HES.^5^ However, based on non‐specific signals of anti‐human herpes viruses 6 (HHV‐6) antibody in his blood sample, we could not clearly rule out drug‐induced hypersensitivity syndrome which is characteristically associated with reactivation of HHV‐6. We diagnosed the case as erythroderma with secondary HE caused by chronic drug eruption, or HES.

As his health insurance did not cover biologics for hospitalized patients, systemic therapy with prednisolone (0.6 mg/kg/day) was initiated. However, no improvements in HE, erythroderma or purpura located on the right toes were seen. A second biopsy was therefore taken from the right toes on Day 11, showing fibrin deposition in peripheral arteries without perivascular inflammatory cells (Figure 1i). This suggested arterial thrombosis rather than vasculitis, although we could not rule out eosinophilic vasculitis. No evidence of cholesterol embolism was identified. Ankle‐brachial index was normal, and computed tomography (CT) did not identify any vessel wall abnormalities in medium to large size vessels in the body. Negative results were obtained for anti‐neutrophil cytoplastic antibody in serum and anti‐nuclear antibody. These and no detection of granuloma in skin pathology gave support to exclude eosinophilic granulomatosis with polyangiitis (EGPA). Clinical and pathological presentation was not compatible with Kimura's disease, which occurs mostly in head and neck and is charactered by dense infiltration of lymphocytes/eosinophils in subcutaneous masses together with blood eosinophilia and high IgE levels.^6^ We did not perform serum IgG4 detection/IgG4 staining of skin biopsy before treatment. However, we excluded IgG4‐related disease depending on no other organ failures, absence of tumour detection in CT, no evidence of fibrosis/plasma cell infiltrations in skin samples. After 2 weeks of systemic prednisolone, eosinophil count suddenly decreased to 0.3 × 10^9^/L, but erythroderma remained and haemorrhagic blisters appeared at the area of purpura on the right hallux.

## 4 Additional treatment

**FIGURE 2:**
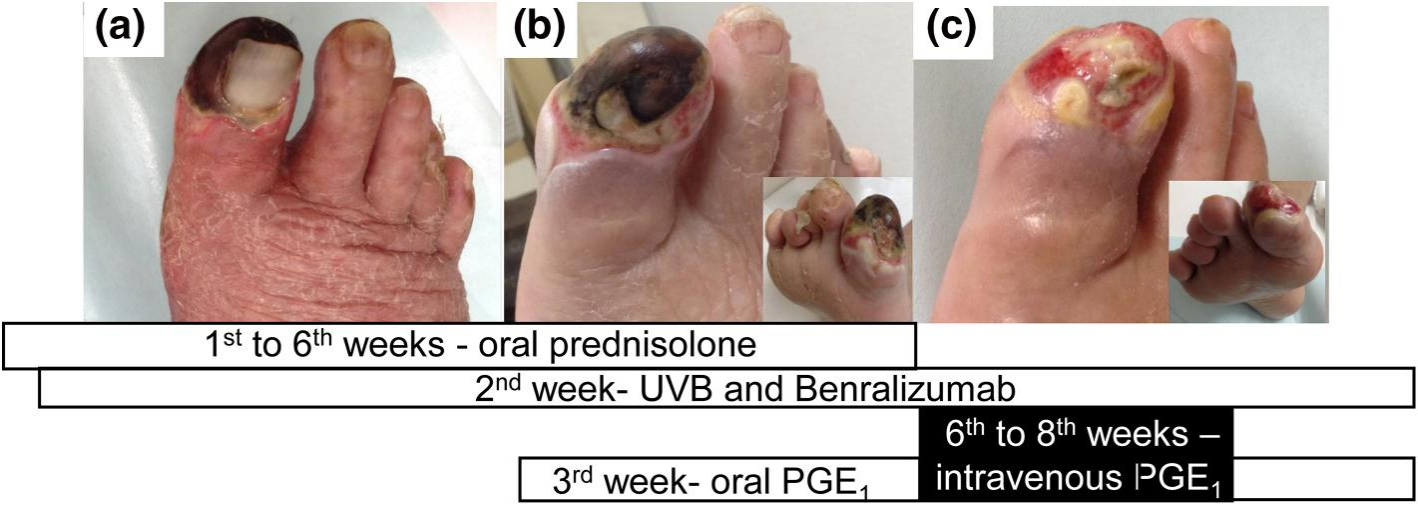
In the 3rd (a), 6th (b) and 12th (c) weeks under treatment with oral prednisolone, benralizumab and NB‐UVB. (a) Necrotic ulcer with peripheral coldness is present on the right hallux, although other skin lesions with purpura gradually resolved from 3rd to 6th weeks. (b) An additional 3 weeks of treatment with oral prostaglandin E_1_ (limaprost alfadex at 15 µg/day) did not rescue digital ischaemia/necrosis. (c) Decreased size of the ulcer and progressive granulation/epithelialization are observed in the right hallux, as a result of treatment with intravenous PGE_1_ (alprostadil 10 µg/day × 2 weeks) and surgical treatment. The nail has not recovered so far

From the 2nd week, additional therapies were initiated in the outpatient clinic for possible eosinophilic inflammation and vasculitis of the skin/vessels: (1) subcutaneous injection of benralizumab (30 mg), humanized monoclonal antibody against interleukin (IL)‐5 receptor α, at 1‐month intervals; and (2) narrow‐band ultraviolet B (NB‐UVB) therapy 3–5 times/week. Erythema began to disappear gradually and eosinophil count became 0/L shortly after administrations of these treatments. The dosage of prednisolone was tapered, then terminated after the 6th week. However, as a necrotic ulcer that appeared from the 3rd week on the right hallux proved resistant to these medications (Figure 2a​), oral prostaglandin (PG)E_1_, with known treatment effects of vasodilation and inhibition of platelet aggregation, was started from the 3rd week. Additional interventions were needed: (1) intravenous PGE_1_ from 6th to 8th weeks, in place of the oral PGE_1_; (2) surgical excision of necrotic tissue in the 12th week (Figure 2b); and (3) excision of sequestrum in the 23rd week. Using these treatments, the right hallux escaped amputation (Figure 2c).

NB‐UVB, which had been provided to a total of 30.6 J/cm^2^, was ended in the 10th week. Benralizumab, at 1‐month interval from the 1st through third injection and 2‐month interval from the fourth injection, kept eosinophils at an undetectable level for more than 3 months with successful control of asthma and eczema.

## 5 Discussion

Eosinophil‐related organ damage involves fibrosis (lungs, heart, digestive tract and others), thrombosis or thromboembolism, neuropathy and others.^1^ From another perspective, ‘idiopathic eosinophilic vasculitis’ is also referred to in asthma‐free patients with HES.^7^ Digital ischaemia with an unproven trigger has previously been reported as a rare initial symptom of HES. Rohmer et al.^8^ presented 33 cases of distal ischaemia related to HES in the literature. Within these cases, vascular involvement appeared in the upper limb in 79%, lower limb in 64% and both in 42%. Amputations were needed in 24% of cases.^8^

Since IL‐5 is a key cytokine for priming and survival of mature eosinophils, IL‐5‐targeting therapies like benralizumab^9^ and mepolizumab^10^ ^,^ ^11^ are increasingly used to treat eosinophilic disorders, including HES. However, benralizumab did not affect digital ischaemia in our case. Of note, intravenous PGE_1_ was essential for our patient. If he had been treated earlier with intravenous PGE_1_, he might have recovered from digital ischaemia earlier. Simple differentiation between thrombosis and vasculitis in HE‐related purpura is difficult, because vasculitis can be transient or episodic, particularly under treatments for ongoing multiple symptoms in HE/HES. However, the present case highlights the importance of earlier intervention for digital ischaemia in the therapeutic management of HE/HES.

## CONFLICT OF INTERESTS

The authors declare that there are no conflict of interests.
